# Binary Sand Cat Swarm Optimization Algorithm for Wrapper Feature Selection on Biological Data

**DOI:** 10.3390/biomimetics8030310

**Published:** 2023-07-14

**Authors:** Amir Seyyedabbasi

**Affiliations:** Software Engineering Department, Faculty of Engineering and Natural Science, Istinye University, 34396 Istanbul, Turkey; amir.seyyedabbasi@istinye.edu.tr

**Keywords:** binary sand cat swarm optimization, metaheuristic algorithm, feature selection, biological data, optimization problems, classification

## Abstract

In large datasets, irrelevant, redundant, and noisy attributes are often present. These attributes can have a negative impact on the classification model accuracy. Therefore, feature selection is an effective pre-processing step intended to enhance the classification performance by choosing a small number of relevant or significant features. It is important to note that due to the NP-hard characteristics of feature selection, the search agent can become trapped in the local optima, which is extremely costly in terms of time and complexity. To solve these problems, an efficient and effective global search method is needed. Sand cat swarm optimization (SCSO) is a newly introduced metaheuristic algorithm that solves global optimization algorithms. Nevertheless, the SCSO algorithm is recommended for continuous problems. bSCSO is a binary version of the SCSO algorithm proposed here for the analysis and solution of discrete problems such as wrapper feature selection in biological data. It was evaluated on ten well-known biological datasets to determine the effectiveness of the bSCSO algorithm. Moreover, the proposed algorithm was compared to four recent binary optimization algorithms to determine which algorithm had better efficiency. A number of findings demonstrated the superiority of the proposed approach both in terms of high prediction accuracy and small feature sizes.

## 1. Introduction

Recently, several metaheuristic algorithms were introduced to solve global optimization problems [1,2,3]. It is well known that these algorithms can be used in optimization [4,5,6]. As a problem becomes larger, the computation time and cost will also increase. Classical mathematical methods cannot be utilized to solve these problems due to the complexity of the problems. Using approximate approaches such as metaheuristic algorithms to solve NP-hard problems (uncertain polynomial time) might be a way to handle NP-hard problems (uncertain polynomial time) as well [7]. These algorithms can solve complex problems in a reasonable time. A metaheuristic algorithm is designed to find near-optimal solutions to high-dimension complex problems because the search space expands as the dimensions increase. In general, most metaheuristic algorithms resolve problems in an effective and efficient manner to achieve near-optimal results. As a result of the significant increase in optimization algorithms in recent years [8,9], there is now an abundance of optimization algorithms that are either intended to improve algorithms or to improve their disadvantages.

Metaheuristic algorithms are divided into four categories: evolution-based, swarm intelligence-based, physics-based, and human-based [7]. The evolution-based algorithms are based on the evolutionary behavior of creatures. Some of the well-known algorithms in this category include the genetic algorithm (GA) [10], differential evolution (DE) [11], evolutionary programming (EP) [12], and biogeography-based optimization (BBO) [13]. The Swarm Intelligence (SI) approach mimics animals’ collective behavior [7]. Popular algorithms in this category include particle swarm optimization (PSO) [14], grey wolf optimization (GWO) [15], the bat algorithm (BA) [16], and sand cat swarm optimization (SCSO) [9]. Physics-based algorithms are influenced by the physical rules of nature. The most famous algorithms in this category consist of black hole (BH) [17], atom search optimization (ASO) [18], big bang–big crunch (BBBC) [19], and simulated annealing (SA) [20]. Human activities with evolution-based processing are modeled mathematically by human-based metaheuristic algorithms. Well-known algorithms in this category include Tabu (Taboo) Search (TS) [21], Teaching–Learning-Based Optimization (TLBO) [22], Variable Neighborhood Search [23], GRASP [24], and Iterated Local Search [25]. The sand cat swarm optimization (SCSO) algorithm is one of the new metaheuristic algorithms for continuous optimization problems [9]. The SCSO algorithm is based on sand cat behavior. This algorithm uses sand cats as search agents in continuous real search spaces to find near-optimal solutions. The SCSO algorithm is described in detail in the next section. Compared with a newly proposed metaheuristic algorithm, the SCSO algorithm has remarkable performance. Based on the no-free-lunch (NFL) theorem [6], there is no algorithm suitable for all problems. In this way, each metaheuristic algorithm may be suitable for some problems and find optimal solutions. Metaheuristic algorithms are used in a wide variety of industries, including health care, engineering, biology, and finance [26,27,28,29,30].

The feature selection (FS) technique is one of the most popular dimension reduction techniques [31]. This technique eliminates the redundant and noisy attributes while selecting only the relevant ones. Using a set of significant features from a large dataset is advantageous not only in terms of efficiency, but also in terms of computational complexity; thereby, enhancing the classification accuracy. It is well known that FS methods have been used in many studies over the years. FS algorithms can be divided into three major categories: filters, wrappers, and embedded approaches [32]. This classification was made through a learning algorithm (classifier) which was a learning algorithm (classifier). Filter-based FS methods remove the irrelevant features from data based on the statistical characteristics of the data. There are many popular filter approaches, such as information gain, *t*-test, chi-squared test, and correlation-based feature selection to select features, all of which can be used in a filtering process. In FS methods using wrappers, a specific machine learning algorithm is used to reduce a subset of the data and evaluate it. As part of the learning algorithms training, these methods employ Cross-Validation (CV) schemas [33]. A significant characteristic of this type of approach is that the learning algorithm and feature selection are tightly coupled together. In this type of approach, the feature selection algorithm is one of the main sections of the learning algorithm. Metaheuristic algorithms are widely used to find an optimal solution to NP-hard problems. The feature selection problem is one of the problems to be solved in this case. In recent years, several metaheuristic algorithms have been introduced that reduce medical data [34].

An approach based on wrappers is used to reduce the feature selection issues using the Binary Golden Eagle Optimizer with Time Variable Flight Length (BGEO-TVFL). As a result of BGEO-TVFL, binary GEO exploration and exploitation are balanced by time-varying flight lengths [35]. The authors proposed a binary Coronavirus Disease Optimization Algorithm (BCOVIDOA) to select the features, a mechanism that mimics the Coronavirus replication mechanisms when hijacking human cells [36]. For evaluating the performance of the proposed algorithm, benchmark datasets from the UCI Repository were utilized. It was proposed [37] to develop a wrapper-based Binary Improved Grey Wolf Optimizer (BIGWO) to categorize Parkinson’s disease with optimal features. In this study, five different transfer functions were used to encode the search space for features. Additionally, the BIGWO algorithm was evaluated for classification performance with adaptive kNN (AkNN). One of the recently introduced metaheuristic algorithms known as the Marine Predator Algorithm (MPA) successfully solved optimization problems [38]. This study aimed to find the optimal subset of features in the datasets using a novel Binary Marine Predator Algorithm (BMPA-TVSinV). A continuous search space was converted to a binary one using two new time-varying transfer functions.

The authors of [39] have presented the binary version of the ant lion optimization algorithm. In addition, they have presented the binary version of the GWO algorithm used in the feature selection problem [32]. Due to the increase in metaheuristic algorithms, the number of binary versions of algorithms has increased. A version of the cockroach swarm optimization was proposed in [40]. The binary bat algorithm was proposed in [41]. The authors of this study used the Sigmoid transfer function to adopt binary algorithms for discrete optimization problems. There is a binary version of the WOA algorithm described in [42]. The authors of this study presented a binary version of WOA to predict photovoltaic cell parameters. According to another study [43], a new binary version of the WOA algorithm was proposed for solving marketing problems. Using the S-shaped transform function, another binary version of the WOA algorithm was proposed in [44].

For the purpose of selecting the most appropriate features for the COVID-19 dataset, a hyper-learning binary decision algorithm (HLBDA) [45] has been introduced. Hyper-learning is employed in this strategy to learn dragonflies based on the top solutions available on a global and personal basis. Ref [46] demonstrates that NSGA II is an effective method for selecting the potential features to be considered. It has recently been suggested that a fast rival genetic algorithm might be an effective solution to the FS problems [47]. The proposed method was demonstrated to find the informative feature subset and was able to do so in a short time compared to conventional methods. Another work [48] has been published proposing a wrapper-based binary SCA (WBSCA) based on a V-shaped transfer function. Using both S-shaped and V-shaped transfer functions, the binary butterfly optimization algorithm (bBOA) addresses the feature selection issues. The bBOA model fails to balance exploration and exploitation. The local search strategy involves butterflies only changing positions randomly, which is considered inadequate [49].

Sentiment classification was improved with the use of the iterated greedy metaheuristic. This was employed by [50] to select quality features for improving its performance. As a method for feature selection in [51], the authors combined the whale optimization algorithm with simulated annealing techniques. It can be concluded that the hybrid approach that they used utilized the simulated annealing method to enhance their search agents’ exploitation ability in promising areas. This was carried out by enhancing their search capabilities. The S-shaped and V-shaped transfer functions were used in this study to develop a binary EPO (BEPO) algorithm [52]. In this algorithm, the V-shaped transfer function is more efficient than the S-shaped. In [53], one of the most recent metaheuristic algorithms for tackling this problem has been applied to feature selection. 

It was discovered that the Hamming distance based BPSO algorithm (HDBPSO) can be used to operate on high-dimension datasets [54]. A local search algorithm was developed in [55] to facilitate the selection of minimal reductions in the PSO algorithm that was based on the correlation information provided by the correlation function. This study proposes a new binary version of the Crow search algorithm (CSA) algorithm named bSCA [56]. The bCSA is binarized using a sigmoid transformation. In this study, the proposed algorithm was used to solve a two-dimensional bin packing problem. In order to select subgraphs with the highest accuracy, a binary cat swarm intelligence technique was applied at each level of classification [57]. The binary cat swarm intelligence technique ensures the most accurate subgraphs are selected for classification. It also improves the overall accuracy and speed of classification. This paper presented an improved binary version of the SSA based on a modified Arctan transformation [58].

The proposed algorithm was evaluated on benchmark datasets and compared to other existing methods. The results showed that the proposed method outperformed the existing methods in terms of accuracy and execution time. Regarding the transfer function, this modification possessed two characteristics: multiplicity and mobility. It was possible to enhance the exploration and exploitation capabilities by making this modification. This paper presents a hybrid approach consisting of a new multi-objective binary chimp optimization algorithm (MOBChOA) and a deep convolutional neural network (DCNN) for feature selection [59]. MOBChOA and DCNN were used in combination to select the most relevant features and optimize the hyperparameters for image classification. The results of this approach were evaluated and compared with the existing methods.

The following contributions were made as a result of the need for novel and efficient optimization algorithms:

- An innovative binary version of the sand cat swarm optimization algorithm was presented.
- Binarization of the sand cat swarm optimization algorithm was achieved using the V-shaped transfer function.
- An extensive evaluation of the bSCSO’s performance was conducted against a set of 10 well-known biological benchmarks.
- A comparison was made between the bSCSO algorithm and the well-known binary metaheuristic algorithms.

The remainder of the paper is organized as follows. The Section 2 describes the sand cat swarm optimization (SCSO) algorithm. The proposed binary sand cat swarm optimization algorithm (bSCSO) is described in more detail in the Section 3. In the Section 4, a discussion and analysis of the results are presented. The study’s conclusion is presented in the Section 5 of the report. 

## 2. Sand Cat Swarm Optimization (SCSO) Algorithm

There is a metaheuristic algorithm called sand cat swarm optimization (SCSO), inspired by the sand cats’ behavior in nature [9]. Sand cats can hear sounds below 2 kHz. In contrast to domestic cats, sand cats prefer sandy and stony deserts. In terms of appearance, there is not a significant difference between these two types of cat. Due to the harsh conditions in their living environment, sand cats’ soles and palms are entirely covered with fur. This gives them protection against heat and cold. It is also difficult to track cats’ footprints due to this characteristic. As mentioned above, the sand cat’s ability to detect low-frequency noises makes its ears the most distinctive aspect of the animal. Foraging in a harsh environment is hard for animals, especially small animals. Sand cats hunt during the cool nights and rest underground during the day. They have a different hunting method.

The sand cat has a very special foraging and hunting mechanism. The ability of these animals to locate prey underground or on the ground is the basis for their remarkable ability to locate prey. As a result, they can find their prey quickly. The swarm optimization algorithm (SCSO) imitated this feature to find the most optimal solution [9]. As with other metaheuristic algorithms, the first step is population initialization. The search space is populated randomly based on the problem’s lower and upper boundaries. Each row of search space indicates a search agent solution to a predefined optimization problem. The search agent is usually defined during the initialization. Metaheuristic algorithms optimize a problem and find a near-optimal solution. In this way, for each optimization problem, a fitness function (cost function) is defined to evaluate the obtained solution. Based on the problem objective, the metaheuristic algorithm guides the solution to the goal. For each solution (search agent), the fitness (cost) determines the next iteration until it reaches the last iteration. The result obtained in the last iteration (which is up to the user) is the most optimal solution. Here, each metaheuristic algorithm mechanism reaches the optimum solution. Generally, the hunting mechanism determines the optimum result.

The SCSO algorithm has a special working principle. After initialization, searching for prey is performed to find the optimum solution. In this way, the sand cat’s ability in the low-frequency noise emission is used. Each search agent has a predefined sensitivity range starting at 2 kHz. In the SCSO algorithm, the $\vec{R_{G}}$ parameter linearly decreases from 2 to 0 (Equation (1)). Where S_M_ is assumed to be 2, Iter_c_ is the current iteration number, and iter_max_ is the maximum number of iterations. In this way, in the initial iterations, the sand cat moves quickly and after half of the iterations, its movement becomes more intelligent. As with other metaheuristic algorithms, the trade-off between the exploration and exploitation phases is important; in this way, the SCSO uses a $\vec{R}$ parameter. In accordance with (Equation (2)), the transition between the two phases is balanced. Furthermore, Equation (3) is defined in order to avoid trapping in the local optimum. The $\vec{r}$ parameter determines the sensitivity range of each search agent. The main step of the SCSO is a position update for each search agent. Based on Equation (4), the position update for the corresponding search agent in each iteration is based on the best candidate position and its current position beside the sensitivity range. In Equation (4), the $\vec{pos_{bc}}$, $\vec{pos_{c}}$, and $\vec{r}$ indicates the best-candidate position, current position, and sensitivity range, respectively. After searching for prey (exploration), the next step in the SCSO is attacking the prey (exploitation) phase. The distance between the best position and the current position of each search agent in the corresponding iteration is calculated using Equation (5). As aforementioned, the sand cats’ precise sensitivity is used to hunt their prey. The sensitivity range is assumed to be circular, so in each movement, the direction is determined by a random θ angle based on a roulette wheel selection in the SCSO. A θ random angle between 0 and 360 results in a cosine of between −1 to 1. In this way, a circular movement is achieved. In Equation (5) (in the paper), the $\vec{pos_{b}}$ and $\vec{pos_{rnd}}$ are the best position (best solution) and random position, respectively.

(1) $$\vec{r_{G}}=S_{M}-\left(\frac{S_{M}\times {iter}_{c}}{{iter}_{Max}}\right)$$

(2) $$\vec{R}=2\times \vec{r_{G}}\times rand\left(0,1\right)-\vec{r_{G}}$$

(3) $$\vec{r}=\vec{r_{G}}\times rand\left(0,1\right)$$

(4) $$\vec{Pos}\left(t+1\right)=\vec{r}.\left(\vec{{Pos}_{bc}}\left(t\right)-rand\left(0,1\right)\cdot \vec{{Pos}_{c}}\left(t\right)\right)$$

$$\vec{{Pos}_{rnd}}=\left|rand\left(0,1\right)\cdot \vec{{Pos}_{b}}\left(t\right)-\vec{{Pos}_{c}}\left(t\right)\right|$$

(5) $$\vec{Pos}\left(t+1\right)=\vec{{Pos}_{b}}\left(t\right)-\vec{r}.\vec{{Pos}_{rnd}}\cdot \cos\left(\theta \right)$$

(6) $$\vec{X}\left(t+1\right)=\left\{\begin{array}{c} \vec{{Pos}_{b}}\left(t\right)-\vec{{Pos}_{rnd}}\cdot \cos\left(\theta \right).\vec{r}\\ \vec{r}.\left(\vec{{Pos}_{bc}}\left(t\right)-rand\left(0,1\right)\cdot \vec{{Pos}_{c}}\left(t\right)\right) \end{array}\right.\;\begin{array}{c} \left|R\right|\leq 1;exploitation\\ \left|R\right|>1;exploration \end{array}$$

## 3. Binary Sand Cat Swarm Optimization (bSCSO) Algorithm

In the field of optimization, problems in the binary space are addressed. It is therefore necessary to implement binary versions of metaheuristic algorithms. Typically, in this type of algorithm, the search space includes one or zero, as well as the search agents’ movement in the binary space. The search space is arranged in rows that determine the solution, which is a combination of the binary values for each row. Comparing the binary (discrete) version of each metaheuristic algorithm with the continuous metaheuristic algorithm, the main difference is the particle movements, where zero changes to one and vice versa. In the SCSO algorithm [6], the search space is populated by continuous and real numbers, so in a binary optimization problem, this algorithm cannot be used. As a result of this, this study proposed the binary sand cat swarm optimization (bSCSO) algorithm to solve this problem. bSCSO is an algorithm that has been proposed specifically for binary search spaces. During the position update of each sand cat (search agent), a V-shaped transformation is applied to transfer the obtained values to a range of values between 0 and 1. A solution to the problem is the location of each sand in a 0’s and 1’s vector.

Each sand cat in the bSCSO algorithm detected sounds below 2 Khz, similar to the SCSO algorithm. This method followed the SCSO algorithm, but the search agent moved in the range of [0, 1]. Using Equation (7), each search agent (sand cat) could update its position [39]. In the end, the V-shaped transfer function transfered the result to zero or one. The bSCSO algorithm used the V-shaped function as its main rule. As discussed earlier, the search was performed in a populated search space of zero or one. The lower and upper boundaries were zero and one. After initialization, the search agents’ positions should be updated. Accordingly, using the SCSO algorithm, the sand cat’s searching and hunting phases were aided by its unique hearing ability. In each iteration, each search agent obtained a position to update in this way, and the V-shaped transfer function was used to transfer the result to zero and one.

**Figure 1:**
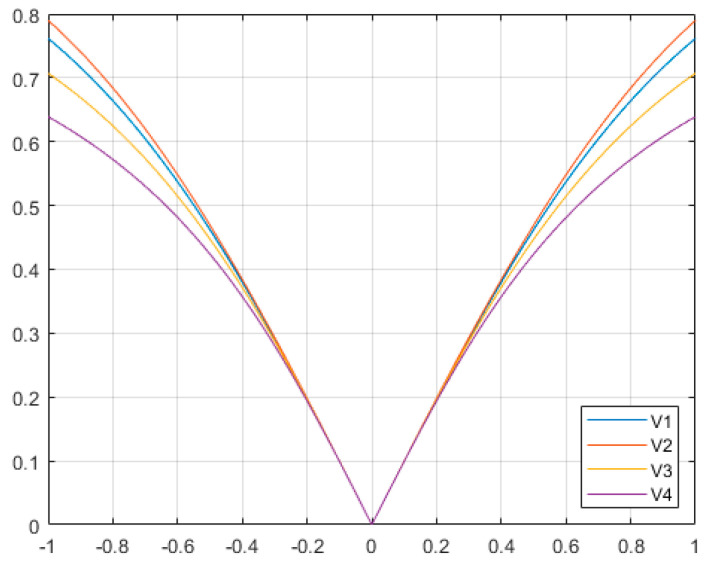
V-shaped transfer functions.

Any value between plus and minus infinity could be transferred to zero or one using a V-shaped transfer function. For each agent in the search space, the obtained result was between zero and one. Additionally, search agents were forced to move in binary space by a rule applied to the bSCSO. The bSCSO transfer function provided a probability of a search agent changing from 0 to 1 and vice versa. The different types of V-shaped transfer functions are described in Table 1. Different types of transfer functions existed here, with a different probability of changing its value. Figure 1 illustrates the four types of V-shaped functions. Exploration and exploitation were affected by the abrupt change between 0 and 1 in a v-shaped function. This behavior enabled the algorithm to explore and exploit the environment simultaneously. It also enabled the algorithm to explore and exploit the environment more efficiently, resulting in improved performance.

**Figure 2:**
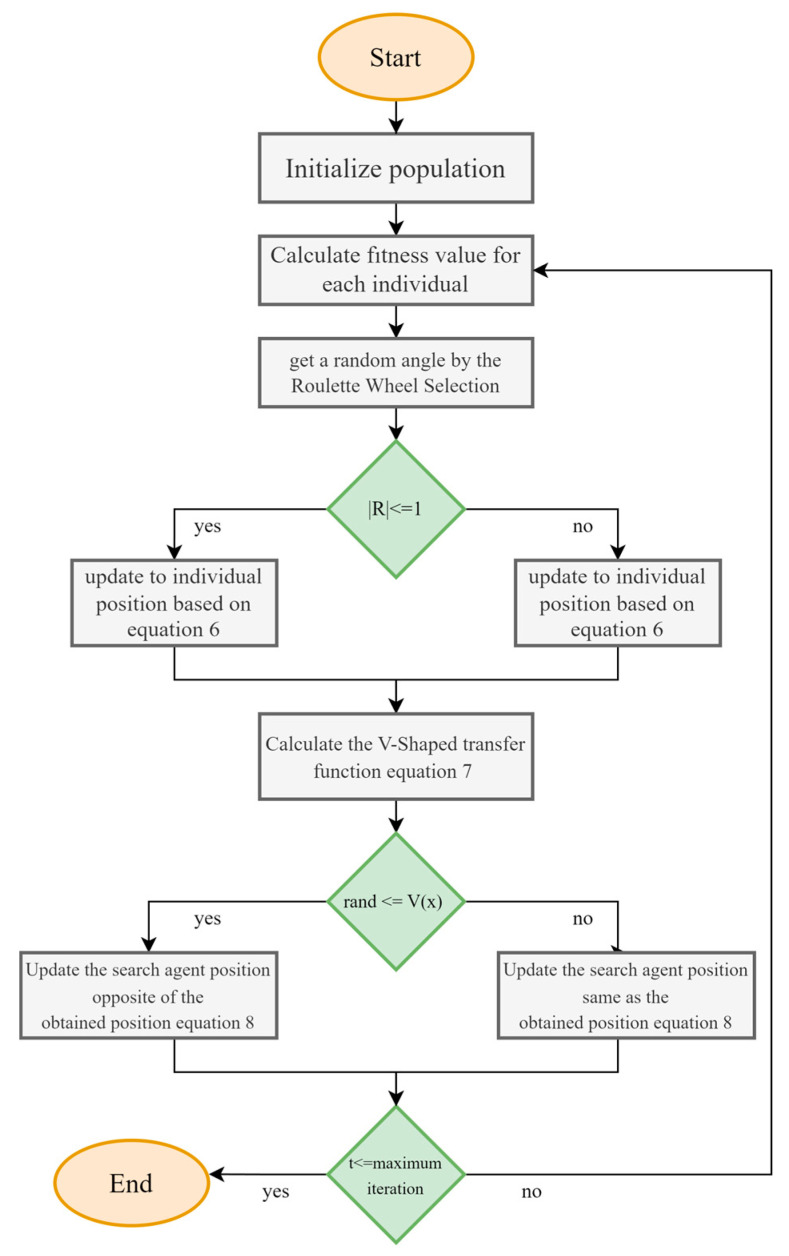
The flowchart of the bSCSO.

In order to achieve the goal, type four of the V-shaped transfer functions and an updating rule for the position were performed, where the $x_{i}^{n}\left(t\right)$ value referred to the location of i_th_ search agent in the n_th_ dimension at iteration t. The rand (random number) was a uniform random number between 0 and 1. Algorithm 1 and Figure 2 provided the pseudocode and flowchart of the bSCSO algorithm.

(7) $$V\left(x_{i}^{n}\left(t\right)\right)=\frac{2}{\pi }\arctan\left(\frac{\pi }{2}x_{i}^{n}\left(t\right)\right)$$

(8) xint+1=(xin)−1(xin)              if rand<Vxintotherwise

**Algorithm 1.** Binary Sand cat swarm optimization algorithm pseudocode.Initialize the population Calculate the fitness function based on the objective function Initialize the r, r_G_, R 
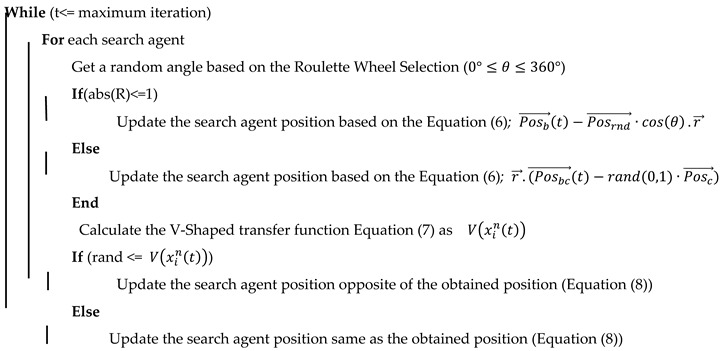
          **End**      **End**   t=t++ **End**

## 4. Simulation and Result Analysis

Feature selection is one of the most frequently encountered problems in computer science, where the search space is a n-dimensional Boolean array. Thus, it can be concluded that the bSCSO algorithm we proposed here could effectively solve the feature selection problems. It should be noted that as the search agent position was determined by selecting or excluding the features, binary vectors were used for expressing the position as ‘1’, which indicated that the feature corresponding to the search agent position was selected, and ‘0’, which indicated that the feature was not selected. Feature selection processes are concerned with maximizing classification accuracy and minimizing the number of features. The bSCSO algorithm took into account these two objectives during its adaptive search to find the combination of features most appropriate for the application. It is important to note that the bSCSO applied a fitness function to search the agent position in Equation (9):

(9) $$fitness=\alpha *ER+\beta \frac{\left|S\right|}{\left|C\right|}$$

The error rate *ER* was defined as the ratio between the number of instances wrongly classified and the total number of instances. Here, *S* was the length of the feature subset, *C* was the total number of features, and B was the number of instances incorrectly classified. It was assumed that the parameters α and β were the weight vectors for determining the importance of classification performance and feature size.

### 4.1. Simulation Setting

This study aimed to enhance learning capabilities, reduce computation complexity, and improve computational efficiency by selecting the relevant features from a dataset to enhance classification performance. We determined the optimal set of features by using a binary algorithm, based upon the nature of the task at hand and upon the characteristics of the subset of features to be selected. Accordingly, each solution was represented by a binary vector of D entries that reflected the number of features in the dataset. As with the binary method, each solution was represented by a binary vector. It is worth noting that there were two entries in the solution vector; one signified the absence of selection, and the other signified the selection of a particular feature. 

A binary SCSO (bSCSO) version was used to assist in the solution of feature selection problems. The performance of different optimization algorithms based on fitness functions was compared using medical datasets to determine which algorithm performed the most efficiently as a result of the comparison. As an evaluation of the accuracy of the combined bSCSO and KNN methods, a series of repeated experiments were conducted (repeated five times to avoid bias). We conducted a comparison of bSCSO’s performance with BMNABC [60], BBA [41], bGA [48], and bPSO [61] in order to determine which performed better. It was based on the accuracy of the data that the study’s results were reported. To measure the effectiveness of the proposed bSCSO, four evaluation metrics were calculated. These metrics included the mean and standard deviation of accuracy and the mean and standard deviation of the selected features.

The algorithm’s simulation parameters are summarized in Table 2. The proposed bSCSO algorithm was evaluated on 10 datasets available in the UCI machine learning repository. Table 3 provides information on the number of features and data objects in each of the medical datasets. In addition, it provides details on the number of classes within each dataset. As part of the evaluation of the performance of the proposed bSCSO algorithm in comparison with other optimization algorithms, we used the same population size of 30. We compared it with the other algorithms in a number of iterations of 100 to test its performance. We also analyzed the performance of each algorithm based on the average value of each objective function over five independent runs. On a computer with a 1.60 GHz CPU and 8 GB of RAM, simulation and analysis were conducted using MATLAB 2020b.

### 4.2. Dataset

A few datasets were selected from well-known dataset repositories such as Heart, Heart-Statlog, Parkinson, Wisconsin Diagnostic Breast Cancer (Wdbc), Breast Cancer, Dermatology, and Lung Cancer. In the feature selection problem, one of the greatest challenges is the analysis of datasets with a high-dimensional feature set. This is followed by the selection of features based on only a few samples. PersonGait, Colon tumor, and Leukemia-3c are examples of high-dimensional datasets. In Table 2, we have provided the names, number of features, instances, and classes of each feature. As can be seen, aside from being one of the most famous and complex medical datasets, the Wisconsin Diagnostic Breast Cancer (Wdbc) is also among the most widely used.

### 4.3. Results and Discussion

There were several datasets chosen to represent the different types of issues based on the instances and attributes associated with them. For cross-validation, each dataset was divided into three randomly selected subsets, including training, testing, and validation data sets. In the case of feature selection, we used the K-NN classifier as a wrapper method to select the features. For this example, we applied K-NN with 3,5, and 7. Each search agent position generated a different subset of attributes as a result of the training process it went through. KNN classifiers were evaluated on the validation subset using the training set during optimization. It was also part of the scope of the bSCSO’s role to provide guidance during the feature selection process. This was in addition to the feature selection. In addition, the optimization algorithm did not know about the test subset at the time of optimization.

This experiment aimed to produce optimal performance by partitioning the data into training and testing sets with a ratio of 8:2. This was so the data could be used to optimize performance. It meant that 80 percent of each dataset was used for training and 20 percent for testing. An evaluation of the proposed algorithm’s performance was conducted with nine medical datasets in this subsection. The datasets were chosen from the UCI machine learning repository [62,63]. The bSCSO’s efficacy could be evaluated based on the mean and standard deviation of its accuracy, as well as the number of features selected. The results achieved by the algorithms are shown in Table 4 and Table 5 as a representation of their results. Table 4 provide the averages and standard deviations for the accuracy and selected features of the binary version of bSCSO with V-shaped transfer function, and other binary version algorithms, based on five runs of the algorithm. Considering the tables described above, it was evident that the bSCSO provided the most accurate accuracy for the most datasets.

Table 3 includes a statistical analysis of the obtained results from different optimization algorithms on the different datasets. Therefore, it was generally considered that the algorithm that had the highest accuracy rate in terms of its mean value of the accuracy was the best solution. Each algorithm’s mean and standard deviation were compared in terms of accuracy and selected features. Additionally, the various KNN parameters values were chosen to determine their efficiency. Consequently, the bSCSO performed very well when compared with other algorithms tested on the datasets compared to the other algorithms. Table 2 shows the results obtained by running each algorithm five times independently using the same parameter configuration.

It was found that the proposed algorithm, bSCSO, selected the most minimum number of features on all datasets. This was compared with the other algorithms BMNABC, BBA, bGA, and bPSO. In some cases, it was also possible to get good performance using the bPSO algorithms. It was possible to rank the bGA algorithm at the second position on the list. Based on Table 3, it should be noted that the bPSO algorithm came in second in the rankings. This was followed by the bGA algorithm, the BBA algorithm, and the BMNABC algorithm.

In tests on Heart, Parkinson, Dermatology, Breast Cancer, Lung Cancer, Person Gait, Colon Tumor, and Leukemia-3c, the bSCSO outperformed the other algorithms. In terms of the most widely used medical dataset, it is important to note that the Heart dataset not only remains one of the most well-known, but it is also one of the most complex. bSCSO has been found to perform extremely well on the Heart datasets when compared to the other algorithms that have been tested against it. Based on the Heart dataset, the bSCSO algorithm was the most effective when the features were selected in the smallest number. Thus, as a result, it was found that the bSCSO algorithm minimized the number of features better than any other algorithm in this study. The bGA and bPSO algorithms outperformed compared with the other algorithms in the Heart-Statlog dataset. In the Wisconsin Diagnostic Breast Cancer (Wdbc) dataset, the bGA algorithm outperformed compared with the other algorithms. The accuracy of the bSCSO algorithm was significantly better than the other algorithms when processing the datasets with very high features such as Person Gait, Colon Tumor, and LEUKEMIA-3C.

The results achieved by the algorithms are shown in Table 4 in terms of the accuracy for each dataset. Table 5 provides the averages and standard deviations for the selected features of the binary version of bSCSO with V-shaped transfer function, and the other binary version algorithms, based on five runs of the algorithm. Considering the tables described above, it was evident that bSCSO provided the most accurate accuracy for most datasets.

**Figure 3:**
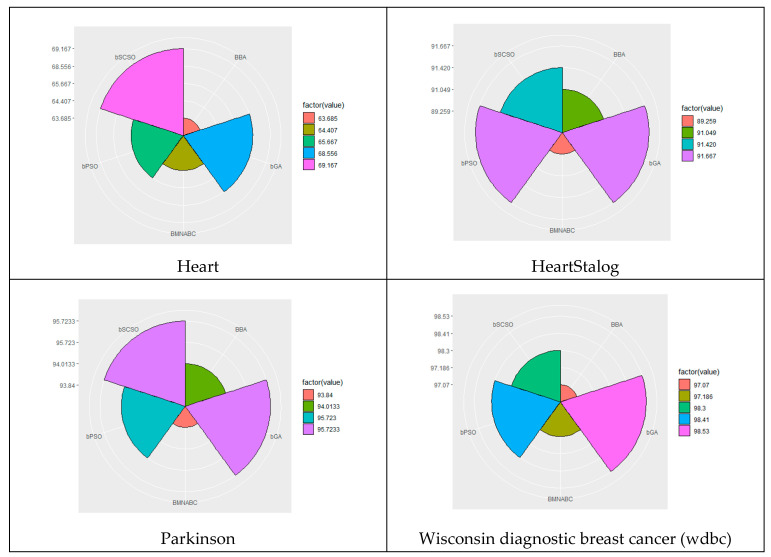

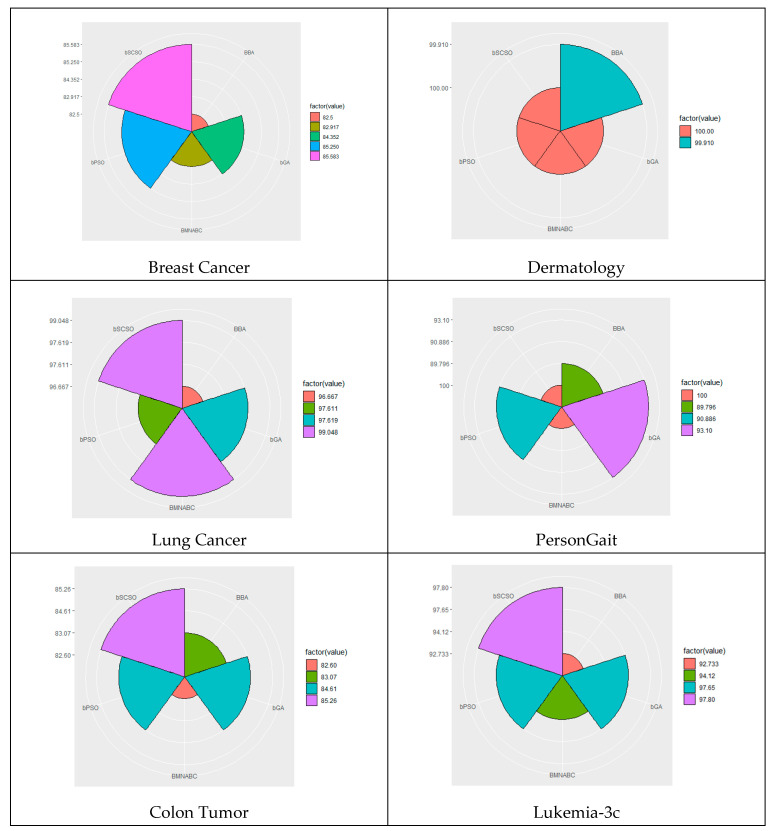
Average of the obtained accuracy for each dataset.

A comparison of the accuracy values for the proposed algorithms and the comparative algorithm for each dataset is shown in Figure 3. It was found that bSCSO, which took into account ten datasets, had the greatest classification accuracy out of all the algorithms. On the other hand, according to this study, the bSCSO only needed fewer features to diagnose a patient’s health. Occasionally, the proposed algorithm was in the second position and had competition to get first place. In addition, no algorithm found the most efficient solution to every problem.

**Figure 4:**
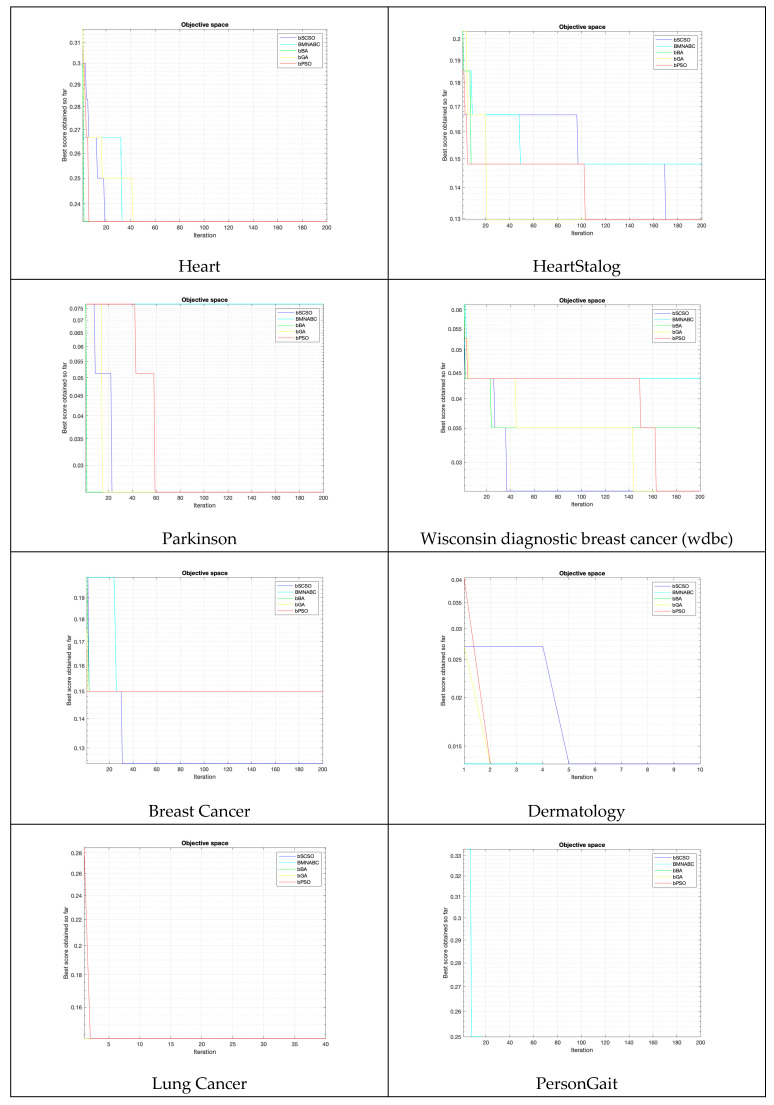

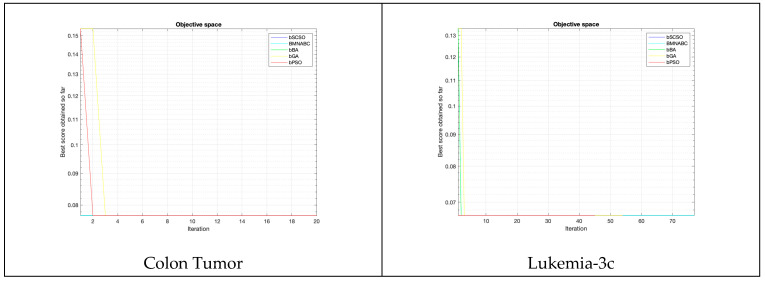
Convergence curves of each dataset.

Figure 4 illustrates the convergence curves for all algorithms on each dataset. The figure shows that the four different algorithms had similar convergence curves for each dataset. This indicated that the algorithms found similar solutions in a similar amount of time, regardless of which dataset they were used on. On different types of data sets, bSCSO performed significantly better than the compared algorithms. The results showed that bSCSO was an efficient and powerful algorithm for solving feature selection problems in biological data. It found optimal solutions quickly and accurately, making it a valuable method for data scientists. In addition, the proposed method performed efficiently. The results indicated that the proposed method outperformed the existing approaches in accuracy and efficiency. Furthermore, it was robust to changes in data and could be applied to different types of problems. The algorithm is suitable for a wide variety of applications, from image processing to robotics and autonomous systems. Its efficiency makes it an attractive choice for data scientists who need optimal solutions.

## 5. Conclusions

This paper proposed a binary version of the sand cat swarm optimization (bSCSO), which was used for the feature selection in wrapper mode. Through the use of V-shaped transfer functions and binary operators, the sand cat swarm optimization (SCSO) was transformed into a binary form so that it could be used as a discrete optimization algorithm. The binary strategy further improved the efficiency of the global and local search by balancing the exploration and exploitation tendencies in order to maximize its efficiency. As a method of evaluating the search performance of a range of algorithms in machine learning, the proposed approaches were used for selecting features to assess their search abilities. This study applied binary algorithms as part of the domain of evaluation. It compared the results with those of well-known feature selection methods, such as bPSO, bGA, BBA, and BMNABC to find out which one performed better. Feature selection is a crucial part of the classification process before classifiers are applied to a set of data to select informative features. To create a high-accuracy classification model at a low computational cost, a feature selection method must be effective.

Feature selection can be designed as a combinatorial problem by using several metaheuristic algorithms, including bPSO, bGA, BBA, and BMNABC. It is fascinating to note that based on the experimental results of bSCSO on a medical dataset, it selected the smallest number of features based on the experimental results. KNN classification could be more accurate with bSCSO, but this algorithm required more time to run. In both the bSCSO and KNN classifications, the lowest number of features was found. As a result, the classifications were more accurate than the other methods. As a result of these experiments, we concluded that the bSCSO algorithm outperformed other similar algorithms. It was more accurate with prediction accuracy as well as minimizing the number of features selected compared to the other algorithms. The performance of the bSCSO algorithm was impressive due to the fact that it allowed the search agents (sand cats) to move in different positions because it used a random angle, which allowed them to move in various positions. Furthermore, it was followed by modified V-shaped transfer functions used to transfer the results into binary values. It is possible to conduct further research on this discrete algorithm by applying a different transfer function to this proposal. In the future, this work is intended to be expanded as follows:

- bSCSO is also applicable to real-world problems and datasets common in the real world.
- The SCSO is particularly suited to applying S-shaped and U-shaped transfer functions.
- The proposed bSCSO can be applied to face recognition and natural language processing problems.

## Figures and Tables

**Table 1:** Variants of V-shaped transfer functions.

Name	Transfer Function
V-Shaped 1	$V\left(x\right)=\left|tanh(x)\right|$
V-Shaped 2	$V\left(x\right)=\left|erf(\frac{\sqrt{\pi }}{2}x)\right|$
V-Shaped 3	$V\left(x\right)=\left|\frac{x}{\sqrt{1+x^{2}}}\right|$
V-Shaped 4	$V\left(x\right)=\left|\frac{2}{\pi }arctan(\frac{\pi }{2}x)\right|$

**Table 2:** The simulation parameters used in each optimization algorithm.

Algorithm	Parameter	Value
bSCSO	**Sensitivity range (*r_G_*)** **Phases control range (*R*)**	**[2, 0]** **[−2r_G_, 2r_G_]**
**BMNABC**	r_min_ r_max_ v_max_	0 1 6
**BBA**	Loudness Pulse rate Frequency minimum Frequency maximum	0.25 0.1 0 2
**bGA**	Crossover rate Mutation rate	0.8 0.3
**bPSO**	c1 c2 W_max_ W_min_ V_max_	2 2 0.9 0.4 6

**Table 3:** Detailed information about the used datasets.

DATASET	FEATURES	DATA OBJECTS	CLASS
**HEART**	13	297	5
**HEART-STATLOG**	13	270	2
**PARKİNSON**	22	195	2
**WİSCONSİN DİAGNOSTİC BREAST CANCER (WDBC)**	31	569	2
**BREAST CANCER**	32	198	2
**DERMATOLOGY**	33	366	6
**LUNG CANCER**	56	32	3
**PERSONGAİT**	321	48	16
**COLON TUMOR**	2000	62	2
**LEUKEMİA-3C**	7129	72	3

**Table 4:** In five independent runs, the following results were obtained in terms of accuracy for each dataset.

Algorithm		bSCSO		BMNABC		BBA		BGA		bPSO	
Dataset	Knn	Mean	Std	Mean	Std	Mean	Std	Mean	Std	Mean	Std
Heart	3	71.66	0	62.66	0.91	61	1.49	71.66	0	63.33	0
	5	68.33	0	63.33	0	61.33	1.82	64.66	0.74	63.33	0
	7	68.33	0	66.66	0	66.66	0	68.33	0	66.66	0
Heart-Statlog	3	92.5926	0	91.4815	1.0143	91.85	1.0143	92.59	0	92.59	0
	5	93.7037	1.0143	82.9630	0.8282	92.96	0.8282	94.44	0	94.4444	0
	7	93.7037	1.0143	92.9630	0.8282	92.96	0.8282	94.44	0	94.4444	0
Parkinson	3	97.43	0	92.30	0	92.30	0	97.43	0	97.43	0
	5	94.87	0	94.35	1.14	94.87	0	94.87	0	94.87	0
	7	94.87	0	94.87	0	94.87	0	94.87	0	94.87	0
Wisconsin Diagnostic Breast Cancer (Wdbc)	3	98.42	0.39	97.54	1.56	97.54	1.56	99.12	0	98.94	0.392
	5	98.24	0	97.36	0.620	98.24	0	98.24	0	98.07	0.39
	7	98.24	0	96.66	1.14	95.43	1.56	98.24	0	98.24	0
Breast Cancer	3	88	0	82.50	2.5	80.50	2.7386	85	0	84.5	1.1180
	5	92	2.7386	88.5	1.3693	89.50	2.73	93.5	2.2361	94.5	1.1180
	7	84.5	2.73	83	1.11	83.50	1.36	80.11	1.77	85	0
Dermatology	3	100	0	100	0	100	0	100	0	100	0
	5	100	0	100	0	100	0	100	0	100	0
	7	100	0	100	0	99.4595	0.7402	100	0	100	0
Lung cancer	3	100	0	97.1429	6.3888	91.4286	7.8246	97.1429	6.3888	100	0
	5	100	0	100	0	94.2857	7.8246	100	0	94.2857	7.8246
	7	94.2857	7.8246	97.1429	6.3888	94.2857	7.8246	88.5714	6.3888	91.4286	7.8246
Person Gait	3	100	0	100	0	90.83	8.53	86.66	7.45	93.33	9.12
	5	100	0	100	0	96.66	7.45	100	0	100	0
	7	100	0	100	0	81.90	16.63	92.66	10.11	79.33	12.50
Colon tumor	3	84.61	0	81.6	0	84.61	0	84.61	0	84.61	0
	5	86.57	0.78	84.61	3.44	83.07	3.44	84.61	0	84.61	0
	7	84.61	0	81.6	0	81.53	4	84.61	0	84.61	0
Leukemia-3c	3	97.65	1.17	94.12	0	93.4	0.47	97.65	0	97.65	0
	5	97.65	0.56	94.12	0	91.4	1.49	97.65	0	97.65	0
	7	98.12	0.78	94.12	0	93.4	0.49	97.65	0	97.65	0

**Table 5:** Each dataset selected features using different nearest neighbor sizes (KNN).

Algorithm		bSCSO		BMNABC		BBA		BGA		bPSO	
Dataset	Knn	Mean	Std	Mean	Std	Mean	Std	Mean	Std	Mean	Std
Heart	3	3	0	4	1.2247	5	0.7071	3.4	0.8944	6.8	1.0954
	5	3	0	2	0	3.8	1.09	4.4	1.3416	2	0
	7	4	0	5	0	5	0	4	0	5	0
Heart-Statlog	3	4	0	3.4	0.5477	4.2	1.0954	4	0	4	0
	5	4.8	1.6432	3.6	1.3416	3.6	1.3416	6	0	6	0
	7	4.8	1.6432	3.6	1.3416	3.6	1.3416	6	0	6	0
Parkinson	3	6	0	3	0	3	0	6.40	0.547	6.60	1.34
	5	2	0	2.40	0.54	3	0	3	0	2.80	0.44
	7	3	0	3	0	3	0	3	0	3	0
Wisconsin Diagnostic Breast Cancer (Wdbc)	3	7.20	1.64	6.20	1.92	6	1.87	13.8	1.095	10.8	1.64
	5	7.20	0.44	5.20	1.09	7.40	0.54	11.40	0.89	9	1.87
	7	7	0	4.8	1.78	3	2.23	10	0	9.8	2.04
Breast Cancer	3	3	0	2.6	0.5477	3.6	0.5477	9.4	1.5166	10	2.7749
	5	3.6	2.7386	1.4	0.6588	2.6	0.8944	11.2	4.0866	9.4	1.5166
	7	1.40	0.547	2.6	0.8944	3	0	10.4	1.1402	9	1
Dermatology	3	9.8	3.1145	8.8	1.3038	9.8	1.3038	30.8	1.7889	12.6	0.5477
	5	9.2	2.7749	7	1.4142	8.8	3.3466	26.4	7.0569	10.4	0.8944
	7	12	1.4142	10.6	0.8944	10.4	0.8944	32.4	0.5477	13.6	2.0736
Lung cancer	3	6	0	5.2	1.0954	6.4	2.0736	20.8	4.6043	21	2.1213
	5	7.6	2.8810	5.4	0.8944	5.2	1.3038	23.4	2.9665	19.8	4.8683
	7	7.4	3.5777	5	1.5811	6	1.5811	18	3.1623	18.2	3.2711
Person Gait	3	91.8	6.54	1.80	1.09	88.8	12.35	145.40	4.56	147.4	4.44
	5	87.8	3.63	2	0	69.80	7.19	144.8	5.93	134.20	6.22
	7	88.1	4.3	1.2	0.44	77.20	4.438	150.60	10.18	141.2	7.56
Colon tumor	3	721.6	18.52	3.4	1.14	738.8	12.59	898.8	6.7	883	10.02
	5	725.8	13.011	2.8	1.7	751.8	16.154	920.8	10.616	893.6	13.72
	7	732.4	19.12	3.2	1.308	755	19.55	950	14.3	906.4	27.64
Leukemia-3c	3	1964.2	66.93	2101	14.3	3033.8	11.96	3324.8	15.57	3283.2	24.58
	5	1971	48.2	2141	41.15	3030.2	19.52	3326	14.61	3297.6	16.89
	7	1969.4	47.1	2200	13.26	3039.1	14.93	3333	14.17	3304	13.76

## Data Availability

The datasets generated during and/or analyzed during the current study are available from the corresponding author on reasonable request.
